# A comparison of spouse and non-spouse carers of people with dementia: a descriptive analysis of Swedish national survey data

**DOI:** 10.1186/s12877-021-02264-0

**Published:** 2021-06-02

**Authors:** Marcus F. Johansson, Kevin J. McKee, Lena Dahlberg, Christine L. Williams, Martina Summer Meranius, Elizabeth Hanson, Lennart Magnusson, Björn Ekman, Lena Marmstål Hammar

**Affiliations:** 1 School of Health and Welfare, Dalarna University, SE-791 88 Falun, Sweden; 2 Aging Research Center, Karolinska Institutet & Stockholm University, Tomtebodavägen 18A, 171 65 Solna, Sweden; 3 Christine E Lynn College of Nursing, Florida Atlantic University, FL 334 31 Boca Raton, USA; 4 School of Health, Care and Social Welfare, Mälardalen University, SE-721 23 Västerås, Sweden; 5 Swedish Family Care Competence Centre, Linnaeus University, Box 762, SE-391 27 Kalmar, Sweden; 6 Department of Clinical Sciences, Lund University, SE-202 13 Malmö, Sweden; 7 Department of Neurobiology, Care Sciences and Society, Karolinska Institutet, Alfred Nobels Allé 23, SE-14152 Huddinge, Sweden

**Keywords:** Informal carer, Spouse carer, Dementia, Care provision, Population study, Sweden

## Abstract

**Background:**

Being an informal carer of a person with dementia (PwD) can have a negative effect on the carer’s health and quality of life, and spouse carers have been found to be especially vulnerable. Yet relatively little is known about the care provided and support received by spouse carers. This study compares spouse carers to other informal carers of PwDs regarding their care provision, the support received and the psychosocial impact of care.

**Methods:**

The study was a cross-sectional questionnaire-based survey of a stratified random sample of the Swedish population aged 18 or over. The questionnaire explored how much care the respondent provided, the support received, and the psychosocial impact of providing care. Of 30,009 people sampled, 11,168 (37.7 %) responded, of whom 330 (2.95 %) were informal carers of a PwD.

**Results:**

In comparison to non-spouse carers, spouse carers provided more care more frequently, did so with less support from family or the local authority, while more frequently experiencing negative impacts on their social life and psychological and physical health. Spouse carers also received more carer support and more frequently experienced a closeness in their relationship with the care-recipient.

**Conclusions:**

Spouse carers of PwD differed from non-spouse carers on virtually all aspects of their care situation. Policy and practice must be more sensitive to how the carer-care-recipient relationship shapes the experience of care, so that support is based on an understanding of the individual carer’s actual needs and preferences rather than on preconceptions drawn from a generalised support model.

**Supplementary Information:**

The online version contains supplementary material available at 10.1186/s12877-021-02264-0.

## Background

In 2015, 46.8 million people globally were living with a dementia condition, with 7.4 million persons with dementia (PwDs) living in Western Europe. With current projections the number of PwDs is expected to almost double every two decades, which will have a severe impact on health and social care services [1, 2]. Over the past decade or so, health and social care providers have faced difficulties in providing adequate care for the increasing number of PwDs, and informal carers account for a substantial proportion of the total care for older adults [3–7]. In Sweden, there have been cutbacks in institutional care for older adults and an increase in informal care over the past decade [8, 9], with the result that nearly half of PwDs reside in ordinary housing and receive little or no formal social care services [6, 10, 11]. While informal carers constitute a heterogenous group of people, they are most often the spouse or an adult child of the care-recipient [12–14]. This study presents the findings from a Swedish national survey of informal carers and compares the situation of spouse carers to other informal carers when caring for a PwD.

Spouse carers have been identified as an especially vulnerable group of informal carers, often being older than other informal carers and also tending to provide more extensive care [15]. A recent review showed that spouse carers of PwDs are at risk of developing several different conditions due to disturbed sleep and chronic stress [16]. Other research shows that spouse carers can experience more negative effects of the caring role compared with other informal carers of PwDs, with a reduced quality of life [17, 18], poorer health and increased rates of depressive symptoms [12], poorer social relationships [19] and a greater amount of grief due to their care-recipient’s illness [20, 21]. However, relatively few studies have had a focus specifically on the associations between the spousal relationship and the carer’s provision of care, the support received and the psychosocial outcomes of their care situation, and studies there have mostly used qualitative methods thus limiting the generalisation of their findings.

Globally, the situation of informal carers of PwD has become a prioritized area [2, 22], and the need for targeted support for informal carers has also been identified as an important issue for the future of dementia care both nationally and internationally [22, 23]. In Sweden, local authorities are obliged to provide support to informal carers. With little national guidelines on how the support should be provided and the principle of local authorities’ self-governance, the organisation of support to carers differs both in content and quality across municipalities [23, 24]. There are also concerns that the content, amount and quality of support are dependent on municipality’s priorities [25]. In the national strategy for improvements in dementia care, the Swedish National Board of Health and Welfare (NBHW) stated that informal carers are an important part of dementia care, that they should be included to a greater degree in the planning and provision of care, and that they are in need of further support in their carer role. The Board also stated that the support currently being offered is rarely targeted at specific carer groups such as spouse carers, nor organised according to their needs. The Board concluded that further research is required in order to develop adequate support that meets the needs of carers of PwD [23, 24]. An important first step for such research would be to identify in what ways spouse carers differ from other carers of PwD in terms of their caring situation and their need of support.

The aim of the present study is to describe the care situation of spouse carers of PwDs in Sweden in comparison to other informal carers by analysing data from a population-based national survey. Our analyses examine how being a spouse carer is associated with their level of care provision, the support they receive and the impact that caring has on their lives.

## Methods

### Design

The study design was a cross-sectional questionnaire-based survey.

### Sampling frame and participants

A stratified random sample of 30,009 of the Swedish population aged 18 years of age or older was drawn by the Swedish national statistics authority Statistics Sweden (Swedish acronym: SCB) from the Total Population Register.

The sample was stratified by region such that an equal number of individuals would be drawn from each region in Sweden. Within the stratified frame, the random sample of individuals was drawn and provided with access to the study questionnaire (web-based or hard copy). Excluding 365 cases (questionnaire returned, wrong address *n* = 316; person not contactable *n* = 49), a total of 11,168 individuals responded to the survey (response rate 37.7 %). Reasons for non-response were: not returned *n* = 17,503; declined participation *n* = 480; prevented from participating *n* = 120; wrong person answered the questionnaire *n* = 195; returned spoiled *n* = 86; promised to send in *n* = 5.

Sample size requirements were determined by the need to recruit enough participants who would meet our criteria for an informal carer (see Data analysis, below) to enable a range of sub-group analyses, such as those for the present analytic sample, i.e., (spouse) informal carers of people with dementia. We based our sample size calculation on the results of a previous Swedish national study [15] and its response rate (55 %). Our own response rate of 37.7 % meant that our sample of informal carers was smaller than anticipated, although still sufficient for most sub-group analyses that we wished to perform to detect moderate-to-small effects, including those for the present analytic sample.

### Material

A questionnaire was developed by the research team (Additional file 1: Apendix 1). An initial point of departure for the research team was a questionnaire developed by NBHW in consultation with experts in survey methodology in the Unit for Measurement Technology at SCB and used in a national study on informal care in 2012 [15]. The data from the 2012 survey was reviewed to determine the performance of individual questions regarding, inter alia, response range and skew, item difficulty (proportion missing responses) and item discrimination, and with data cross-tabulation providing evidence for item convergent and divergent validity. Following this review, to provide comparability of data with the 2012 survey and with reflections on potential improvements to response formats in some cases, all but two of the 37 questions of the original questionnaire were retained as source material for the present questionnaire. Next, the research team considered the coverage of different aspects of informal care in the original questionnaire. Some relatively neglected or omitted topics were identified. As a result, new questions were added on the types of carer support participants had been offered/received, sleep disturbance due to caring, the additional financial costs of care, and the main and secondary reasons the care-recipient required care. In addition, some questions were expanded to provide more detailed data on, e.g., the impact of care on work, the domains of care in which the care-recipient was provided with care and the source of that care, and the number of hours of direct and indirect care provided. Following this revision, and as per the process observed for the 2012 survey, the draft of the questionnaire was submitted for quality appraisal to measurement experts at SCB for review of, inter alia, question wording, response formats, item content (face) validity, and questionnaire navigation (question routing). On receiving feedback from SCB, some minor revisions were made to the questionnaire before proceeding to pilot testing. A convenience sample of family carers was recruited via the Swedish Family Care Competence Centre at Linnaeus University and asked to complete the questionnaire and provide feedback on the relevance, clarity and comprehensibility of the questions, and the ease of navigation of the questionnaire. After reviewing the carers’ data and feedback, minor revisions were made which were given a final review by SCB. At the end of this process, the questionnaire used in the present study had 57 individual questions (28 primary questionnaires with associated sub-questions) situated within four sections: (1) Introduction; (2) Caring and support (general); (3) Caring and support (specific); and (4) Background data. The topics covered in the questionnaire and analysed here, as well as the question wordings and response options are available as an appendix (Additional file 1: Appendix 1).

In the introduction to the questionnaire the study’s definition of a carer was given: those who regularly provide care, help or support to a loved one (to someone in the family, or to someone in a close relationship such as a friend, neighbour or workmate) in a personal capacity due to physical or mental illness, disability or old age. The background information also specified that the survey was not about care provided in the fulfilment of one’s occupation, nor about the care that parents give to their children who have no special needs. A series of examples of regular care, help or support were then provided. Finally, it was specified that only the person to whom the questionnaire had been addressed should complete the questionnaire and do so alone.

Questions on (1) whether the participant regularly gave care and/or support to one or more persons and (2) how often they provided care and support to the person or persons (see additional file 1) were used as filter questions whereby those participants who indicated ‘no’ to the first question or ‘less often than once a month’ to the second question were routed to the last section of the questionnaire (Background data). This was because such participants did not meet the study’s inclusion criteria of an informal carer as someone (a) giving regular care and support and (b) at least once a month.

### Procedure

Questionnaires were distributed by post to the selected potential participants by the Data Collection Department at SCB with instructions that the questionnaire be completed and returned to SCB. For those participants who preferred to complete the questionnaire online, a link to a web-based version of the questionnaire hosted on SCB’s website was provided, where an English language equivalent was also available. Self-completion by post and web were selected as data collection modes for the survey as the length of the questionnaire and sample size combined to make collection via other modes impractical, e.g., face-to-face or telephone interview. While the use of online questionnaires can enhance data reliability through e.g., eliminating errors such as double-responses and making questionnaire navigation easier, people with low-levels of digital competence can be excluded from participation: in Sweden, about 400 000 citizens do not use the internet, the majority older people, especially those aged 76 years or over [26]. In order to promote inclusion and maximise our response rate, we therefore offered options for postal or web-based questionnaire self-completion, this approach also having the advantage of maintaining comparability with the data collection modes used in the NBHW 2012 survey.

The questionnaire was sent to potential participants in October 2018. Up to three reminders were sent, with the last reminder sent in late November. Data collection was closed in early January 2019. Of the participants, 8,370 (74.9 %) completed the postal questionnaire and 2,798 (25.1 %) the web-based questionnaire. Sixty-six participants used the English version of the web-based questionnaire. Postal questionnaires were scanned by the Data Collections Department at SCB and the data merged with data from the web-based questionnaires before being systematically checked for reliability. A standardised approach was taken to detecting, recording and – where appropriate – recoding response errors (e.g., double responses to questions, inconsistencies in responses between questions). Data on gender, year of birth and employment status for each participant were added from SCB’s register, before the cleaned data were entered into an SPSS data file. A technical report on the survey was compiled by SCB and sent together with the anonymised SPSS data file to the research team, where final data reliability checks and cleaning were performed.

In information provided with the questionnaire, participants were informed of the rules for personal data processing contained in the EU data protection regulation, in the Act (2001: 99) and the Ordinance (2001: 100) of the Official Statistics and Public and Secrecy Act; that it was voluntary to participate in the investigation; that responses were being pseudonymised; and that informed consent was required and that by answering the questionnaire and returning it they were providing said informed consent and agreed to their responses being supplemented with background data (described above). Ethical approval for the study was granted by the Regional Ethics Review Board in Linköping (reg.no. 2018/135 − 31).

### Data analysis

IBM SPSS v.26 [27] was used to analyse the data. Survey non-response was unequally distributed by gender, age, being born/not born in Sweden, education, income, civil status, occupation and county. To enhance the representativeness of the sample, the stratified sampling design and non-response bias was compensated for by applying a weight based on a combination of the participant’s region, gender, age and education to all analyses. All data and analyses subsequently reported in this paper are based on the weighted sample.

Participants were first identified in terms of whether they met the study’s inclusion criteria for carer (operational definition in the questionnaire information, then regularly providing care and support to one or more persons at least once a month, see filter questions described above). Of the 11,168 respondents, 1,638 (14.7 %) met the study criteria for being an informal carer. Thereafter, carers of PwDs were selected out for further analysis, *N* = 330 (20.1 % of the sample of carers). Univariate and bivariate analyses as appropriate were performed to examine associations between spouse vs. non-spouse status of carers of PwD with the variables measured in the questionnaire. Specifically, associations were examined in four areas related to the carers’ situation: (1) amount and frequency of care provided, (2) supported and unsupported care provision (3) formal support directed to carers and (4) psychosocial and health outcomes of care. For some variables, response categories were combined to more concisely summarise data or to increase category sample size for analysis. Level of significance for all analyses was set at *p* < .05, due to multiple testing inflating the family-wise error rate each significance test should be considered in the context of the obtained effect size.

## Results

### Sample background characteristics

Just over a fifth (22.5 %) of the carers in our sample of informal carers were spouse carers while approximately half (52.0 %) were adult children caring for a parent, while 14.2 % were siblings or other relatives, 1.3 % parents caring for a child and 7.3 % were acquaintances, neighbours or legal guardians. Nine participants did not indicate their relationship to the care-recipient. A majority of the carers was female (58.6 %), with no significant association between gender and spouse/non-spouse carer status. As would be anticipated, spouse carers were significantly older than other carers (Spouse carers *M* = 73.80, *SD* = 10.65; Non-spouse carers *M* = 52.93, *SD* = 14.08, *t*(319) = -11.80, *p = <* 0.001).

Table 1 presents comparisons between spouse carers and non-spouse carers on background characteristics. There was a significant association between spouse/non-spouse carer status and employment (*χ*^2^(2) = 66.84, *p = <* 0.001): 58.3 % of non-spouse carers were employed, while 70.8 % of spouse carers were retired. Most spouse carers (78.4 %) were co-habiting with their care-recipient, while this was true for only a small minority of non-spouse carers (4.5 %; *φ*(1) = 758, *p = <* 0.001). While the majority of PwDs resided in ordinary housing, there were significant associations between spouse carer/non-spouse carer status and the care recipient’s living arrangements (*χ*^2^(2) = 26.48, *p = <* 0.001), with most of the care-recipients (95.9 %) of spouse carers residing in ordinary housing, while 34.5 % of care-recipients of non-spouse carers resided in either a residential care facility or some form of sheltered or assisted housing.

**Table 1:** Sample characteristics, comparisons of spouse carers vs. other carers (*N* = 330)

		Spouse carer	Non-spouse carer	
Caregiver characteristics		(22.5 %)	(74.8 %)	
**Gender**				*φ*(1) = − 0.008, *p* = .882
Female, %		57.3	58.3	
**Employment status, %**				χ^2^(2) = 66.84, *p* = < 0.001
Employed		13.9	58.3	
Retired		70.8	19.4	
Other		15.3	22.3	
**Co-habitation status, %**				*φ*(1) = − 0.758, *p* = < 0.001
Co-habiting		78.4	4.5	
**Living arrangement, PwD, %**				χ^2^(2) = 26.48, *p* = < 0.001
Ordinary housing		95.9	65.6	
Sheltered or service accommodation		0.0	7.1	
Residential care facility		4.1	27.4	
**Number of people to whom care provided %**				*r*_s_(301) = − 0.27, *p* = < 0.001
One person		97.3	70.0	
Two people		2.7	22.3	
Three people		0.0	5.3	
More than three people		0.0	2.4	
**Hours** **of care in an average week, %**				*r*_s_(293) = 0.54, *p* = < 0.001
< 1 h		5.6	13.1	
1–10 h		21.1	75.8	
11–29 h		25.4	9.0	
30–59 h		28.2	1.2	
60 h or more		19.7	0.8	
**Frequency of care, %**				*r*_s_(299) = − 0.40, *p* = < 0.001
Everyday		74.7	18.7	
At least every week		21.3	61.4	
Once a month or less		4.0	19.9	

*Note*: For analyses in this table *n* varies between 278-322 due to internal missing data

### Amount and frequency of care provided

Table 1 also presents the comparisons for spouse/non-spouse carers for the total amount and frequency of care provided. Spouse/non-spouse status was significantly correlated with number of care-recipients, the majority (97.3 %) of spouse carers provided care for one person while 30.0 % of non-spouse carers provided care for two or more people (*r*_s_(301) = − 0.27, *p* = < 0.001). There was also a significant correlation between spouse/non-spouse carer status and total amount of care provided, with just under half of spouse carers (47.9 %) providing 30 h or more care in an average week while the majority of non-spouse carers (88.9 %) provided care for 10 h or less (*r*_s_(293) = 0.54, *p* = < 0.001). The majority of spouse carers (74.7 %) provided care every day, while this was true for just under a fifth of non-spouse carers (18.7 %, *r*_s_(299) = − 0.40, *p* = < 0.001).

### Supported and unsupported care provision

Across all ten care domains examined, spouse/non-spouse carer status was significantly associated with whether care was provided alone or with the support of other actors (see Table 2). For most care domains, the reported need for care in the care-recipient was proportionately similar whether the carer was a spouse or non-spouse. The only domain with a substantial discrepancy in reported need for care was ‘financial support’, where no need was reported by 49.3 % of spouse carers in comparison to 70.6 % of non-spouse carers (χ^2^(3) = 59.11, *p* = < 0.001). Where there was a perceived need for care, for all but one care domain, the largest proportion of spouse carers indicated that care was provided by them alone, whereas for eight out of the ten care domains the largest proportion of non-spouse carers indicated that care was provided with the help of others. The care domain ‘medications and treatments’ stands out as the domain in which the largest proportion of carers indicated that care was provided by others only (spouse carers 20.0 %; non-spouse carers 54.7 %, χ^2^(3) = 56.91, *p* = < .001).

**Table 2:** Carer support for care-recipient in ten domains of care. (*N* = 330)

		No need of care	I care alone	I care with help from others	Others provide all care	*p*	χ^2^
**Household tasks, %**						< 0.001	56.02
Spouse carers		14.1	49.3	33.8	2.8		
Non-spouse carers		22.6	10.5	49.0	18.0		
**Practical activities, %**						< 0.001	47.41
Spouse carers		5.6	57.7	35.2	1.4		
Non-spouse carers		9.4	17.1	66.9	6.5		
**Physical activity, %**						< 0.001	35.16
Spouse carers		14.3	44.3	40.0	1.4		
Non-spouse carers		17.6	14.3	50.0	18.0		
**Contact with services, %**						< 0.001	27.61
Spouse carers		7.1	51.4	38.6	2.9		
Non-spouse carers		6.7	20.9	59.4	13.0		
**Financial support, %**						< 0.001	59.11
Spouse carers		49.3	39.1	11.6	0.0		
Non-spouse carers		70.6	5.3	14.3	9.8		
**Personal care, %**						< 0.001	63.80
Spouse carers		40.8	31.0	23.9	4.2		
Non-spouse carers		33.3	3.3	30.1	33.3		
**Medications and treatments, %**						< 0.001	56.91
Spouse carers		10.0	45.7	24.3	20.0		
Non-spouse carers		13.6	9.1	22.6	54.7		
**Supervision, %**						< 0.001	68.43
Spouse carers		2.9	50.7	37.7	8.7		
Non-spouse carers		6.5	8.1	74.4	11.0		
**Social relationships, %**						< 0.001	21.94
Spouse carers		13.0	27.5	59.4	0.0		
Non-spouse carers		6.9	8.9	81.7	2.4		
**Cultural activities, %**						< 0.001	34.01
Spouse carers		26.5	39.7	32.4	1.5		
Non-spouse carers		27.5	11.9	42.8	17.8		

*Note*: For analyses in this table *n* varies between 304–317 due to internal missing data; *dfs* for all analyses = 3

There was a significant association between spouse/non-spouse carer status and whether in the view of the carer the needs of the PwD were being met. The largest proportion of spouse carers indicated that the care-recipient’s needs were being met (spouse carers, 54.9 %; non-spouse carers, 42.7 %) while a larger proportion of non-spouse carers (9.8 %) than spouse carers (1.4 %) indicated that they were willing to contribute more care to meet the care-recipient’s needs (χ^2^(2) = 6.87, *p* = .032) (not presented in table).

### Formal support directed to carers

A significant association (*φ*(1) = − 0.187, *p* = < 0.001) was found between spouse/non-spouse carer status and awareness of the provision in the Swedish Social Service Act that requires municipalities to offer support to family carers. Just below half (49.3 %) of spouse carers were aware of the legislation while less than a third (28.3 %) of non-spouse carers indicated the same (not presented in table).

The findings on the level of support offered to carers for ten types of carer support are presented in Table 3. Most types of support had been offered to or received by a minority of carers regardless of spouse/non-spouse status. ‘Information and advice’ was the type of support most offered to or received by carers, with 57.1 % of spouse carers and 24.9 % of non-spouse carers having been offered or in receipt of this support. Almost half of the spouse carers had been offered or received carer group support and just over a third had been offered or had received counselling (38.2 %) or respite care (32.3 %). For six out of the ten types of support, significant associations were found between support offered/received and spouse/non-spouse carer status. For four of these (‘information and advice’, ‘counselling’, ‘carer support group’, ‘respite from caring’), a higher proportion of spouse carers than non-spouse carers had been offered or were receiving support. Few carers had been offered or received the other two types of support (‘keep fit/well activities’, ‘financial benefits/support’), for both the majority of spouse carers had not received the support but were interested in receiving it, whereas the majority of non-spouse carers had not received the support and were not interested in receiving it.

**Table 3:** Carer receipt of or interest in different types of carer support. (*N* = 330)

	Yes, offered/received		No, not offered/received but interested		No, not offered/received not interested			
	Spouse carers	Non-spouse carers	Spouse carers	Non-spouse carers	Spouse carers	Non-spouse carers	*p*	χ^2^
Information and advice, %	57.1	24.9	23.8	43.9	19.0	31.2	< 0.001	23.97
Education, %	21.3	12.5	36.1	43.1	42.6	44.4	0.199	3.23
Counselling, %	38.2	10.3	30.9	30.8	30.9	59.0	< 0.001	28.84
Carer support group, %	45.0	15.0	23.3	24.9	31.7	60.1	< 0.001	27.41
Keep-fit/well activities, %	1.8	6.6	58.9	34.1	39.3	59.4	0.002	12.24
Health check-up/advice, %	11.9	8.2	49.2	36.5	39.0	55.3	0.084	4.95
Financial benefits/support, %	5.1	7.5	59.3	36.7	35.6	55.8	0.007	9.85
Respite from caring, %	32.3	14.5	29.2	15.9	38.5	69.6	< 0.001	21.25
Support via modern technology, %	5.2	3.5	41.4	27.4	53.4	69.0	0.083	4.98
Support that facilitates work, %	5.3	2.7	21.1	32.3	73.7	65.0	0.187	3.36

*Note:* For analyses in this table *n* varies between 278 - 300 due to internal missing data; *dfs* for all analyses = 2

Finally, a larger proportion of spouse carers (31.5 %) than non-spouse carers (6.8 %) indicated that they had received some other kind of support than those specified in the questionnaire (*φ*(1) = − 0.316, *p* = < 0.001) (not presented in table).

### Psychosocial and health outcomes of care

Table 4 presents analysis of the frequency with which carers reported experiencing various forms of negative or positive psychosocial and health outcomes due to providing care. Spouse carers had significantly higher mean scores than non-spouse carers on six out of seven negative outcomes of care: ‘trouble finding time to spend with friends’; ‘struggle to find time to exercise’; experiencing psychological stress; experiencing physical stress; experiencing problems in the relationship with the care recipient and experiencing ‘problems in your relationship with family members’.

**Table 4:** Differences between spouse and non-spouse carers on psychosocial outcomes of care (*N* = 330)

	Spouse carers		Non-spouse carers				
	*M*	*SD*	*M*	*SD*	*t*	*df*	*p*
Trouble finding time to spend with friends	1.49	1.09	0.58	0.74	-6.31	81.06	< 0.001
Struggle to find time for exercise	1.09	1.06	0.54	0.77	-3.92	82.10	< 0.001
Psychologically stressful	1.93	0.98	1.22	0.97	-5.16	298	< 0.001
Physically stressful	1.06	0.97	0.52	0.82	-4.46	293	< 0.001
Problems in relationship with care recipient	1.31	1.04	0.63	0.82	-5.60	90.91	< 0.001
Financial problems	0.33	0.65	0.39	0.87	0.51	290	0.608
Problems in relationship with family members	0.77	0.98	0.51	0.77	-2.24	295	0.026
Experience a sense of satisfaction	1.10	0.95	1.35	1.04	1.81	100.88	0.072
Experience a close relationship with care recipient	2.47	0.87	1.98	1.05	-3.59	304	< 0.001

*Note*. For analyses in this table *n* varies between 292–316 due to internal missing data; range for all analyses = 0–3

For positive outcomes of care, spouse carers had a significantly higher mean score on the frequency of experiencing a close relationship with the care-recipient than non-spouse carers. However, no significant difference was found in mean scores on the frequency of experiencing a sense of satisfaction.

Analyses showed that spouse/non-spouse carer status was significantly correlated with both level of self-reported health and sleep disturbance, with spouse carers having poorer self-reported health and a higher frequency of experiencing sleep disturbance. Since age was previously found to be associated with spouse/non-spouse carer status, and both sleep disturbance and self-reported general health might be anticipated to be associated with age, these analyses were repeated while controlling for participant’s age. The significant associations remained, being a spouse carer was positively correlated with both experiencing more frequent sleep disturbance (*r*_*pb*_(304) = 0.34, *p = < .*001) and with poorer self-reported general health (*r*_*pb*_(304) *= .*31, *p = < .*001) (not presented in table).

## Discussion

 Based on data from a survey of a stratified random sample of the Swedish population, this study examined the situation for spouse carers of PwDs in comparison with other carers of PwDs. The results show that spouse/non-spouse carer status is associated with amount and intensity of care provision, the extent to which care is provided alone or with support, the receipt of carer support, and the experienced negative impact and positive value of care. In comparison to non-spouse carers, spouse carers provide more care more frequently, do so with less support from family or the local authority, while experiencing more frequent restrictions on their social life and negative impacts on their psychological and physical health. Conversely, our results findings indicate that spouse carers in comparison to non-spouse carers receive more carer support and more frequently experience a closeness in their relationship with the care-recipient.

Our study found that spouse carers were significantly older than non-spouse carers and that eight out of ten PwDs were co-resident in ordinary housing with their spouse carer. This reflects the transitioning care of older persons in Sweden where ageing-in-place and marketisation policies have resulted in a reduction in the amount of beds in special housing for older PwDs and where home care services are the pillar of formal care for older adults [28–31]. While being cared for at home in a familiar environment has been shown to be beneficial for the PwD [32, 33], previous research has shown that cohabitation, older age and being a spouse carer are all factors that can contribute to lowered quality of life amongst informal carers [18].

Spouse carers more so than non-spouse carers were aware that they as carers should personally be offered support from local authorities. This finding provides the context for another finding: that being offered/receipt of carer support was associated with spouse/non-spouse carer status for six out of the ten types of support considered in our study. For four of these (‘information and advice’, ‘counselling’, ‘carer support group’, ‘respite from caring’) a high proportion of spouse carers compared to non-spouse carers had been offered/received the support. However, overall the proportion of carers being offered/in receipt of carer support is low, a situation also reported in other studies [34]. In our study, only one form of support – information and advice – was offered to/received by more than a quarter of carers. Furthermore, the profile of which types of carer support were most commonly offered or received was similar for spouse and non-spouse carers. Taken together, these results may suggest that local authorities are targeting spouse carers to a greater extent than non-spouse carers for carer support, but the extent of support is still low and the types of support most commonly offered are similar for spouse and non-spouse carers. This may indicate that local authorities are not sensitive to the different situations of spouse and non-spouse carers when offering support, and thus not providing individualised care to PwDs and their carers as recommended in both national and international dementia care strategies [22, 24, 35].

Our findings regarding the receipt of carer support can be contrasted with the level of support spouse carers received from families and local authorities in providing care to the care-recipient. We found that a significantly higher proportion of spouse carers than non-spouse carers provided care alone across almost all care domains considered in our study. Such a situation should be considered with other findings in our study, such that proportionately more spouse carers than non-spouse carers reported providing care on a daily basis and for over 60 h in an average week. Such findings provide context for the higher frequency with which spouse carers compared to non-spouse carers experienced various negative impacts of providing care. Our findings are supported by those of previous research, in which spouse carers can, depending on the severity of the PwDs’ illness, take on a substantial care responsibility, being preoccupied day and night with providing care and support and neglecting their own interests and needs, and experiencing a lower quality of life [14, 18, 19, 21, 36, 37].

Compared to non-spouse carers, spouse carers more frequently experienced a close relationship to the care recipient due to providing care, yet more frequently experienced difficulties in the relationship with the care recipient. These results may seem contradictory but might be reconciled if considered in the context of the challenges a spouse faces when becoming a carer and adapting to the changes in the nature of the spousal relationship. Previous research has found that due to the condition of the PwD the spouse carer can experience a strain on their relationship as well as lower well-being, but that the carer can adapt to maintain the relationship and in some cases experience a closer relationship with the PwD [19, 20, 38, 39]. While comparing data from national studies is problematic due to methodological as well as policy and cultural differences [34, 40], other national studies have found that spouse carers report higher levels of burden than non-spouse carers [31].

### Study strengths and limitations

A main strength of the present study is its stratified random sample of the Swedish population and the use of a detailed questionnaire to collect data on a wide range of issues relating to carers of PwDs. While the size of the eventual sample of carers of PwD was moderate and the response rate to the survey relatively low, the nature of the sampling frame and the application of weights to adjust for sample stratification and non-response bias mean that we can be relatively confident that our study findings can be generalised to the population. In the full sample, item non-response (internal missing) varied from 1.5 to 15.5 % across questions, non-response being higher among respondents later excluded as non-carers and primarily concerning those respondents completing the postal questionnaire. However, respondents completing the web-based questionnaire were, as anticipated, more likely to be inter alia male, younger, and in better health. Thus, providing optional modes of for questionnaire completion meant that sub-groups of the population were not prevented from participating, this increasing the inclusivity of the study.

As with all cross-sectional studies, one is limited in the extent to which one can make causal inferences based on our findings. In addition, the questionnaire used was not developed specifically for carers of PwD and in order to keep the survey to a reasonable length more detailed questions on, for example, the symptoms of the PwD and the duration of the condition could not be included. Furthermore, only two questions measuring positive aspects of care were included in the survey, and only two on the relationship between carer and care-recipient, which means that a more nuanced picture of a spouse carer’s life with a partner with dementia, in which the undoubted frustrations and despair may be tempered by joys and intimacies, is beyond the remit of the present study.

### Implications for future research, policy, and practice

Despite the fact that our study was not designed to provide a finely nuanced picture of spouse carers of PwDs’ perceived need for support or of how such needs might best be met, still our findings offer some indications of how policy and practice might be further developed. Our analyses confirm the findings of other research that spouse carers can be considered a high-need group compared to other carers, given they provide higher levels of care, with less support for providing that care, and with a greater negative impact on their health and social life. While it is encouraging therefore that our findings suggest that local authorities may be targeting spouse carers for carer support, this finding is tempered by others that indicate the extent of carer support is low and that the types of carer support most offered are similar for spouse and non-spouse carers. Our findings show that spouse carers provide care primarily alone, and it is not clear that standard forms of carer support currently address the isolation of their caregiving situation. Firstly, support needs to address the restrictions spouse carers frequently experience on engaging in other activities due to providing care, this could be achieved through offering more flexible forms of respite care that match the needs and preferences of both the spouse carer and the PwD so that they can enjoy time together and time apart pursuing their own interests or preferred leisure activities [41, 42].

Secondly, support needs to address the emotional isolation spouse carers may experience: we found that spouse carers more than non-spouse carers experienced greater difficulties in their relationship with the care-recipient at the same time as experiencing a greater closeness to the care-recipient. Such findings indicate the importance of providers of dementia care focusing on maintaining and strengthening the care relationship, an approach that can increase resilience and promote well-being for both parties [43, 44]. Examples of such support could be home-based respite care with a focus on enriching activities to strengthen relationships between the PWD and their spouse carer [42, 45]; or day centres appropriate not only for people with advanced dementia but also for people with earlier and middle stages of dementia, so that the PwD is stimulated while the carer also receives proactive respite at a timely point so as to prevent an accumulation of the negative impacts of caring [35]. Lastly, the finding that spouse carers more than non-spouse carers experienced greater difficulties in relationships with other family members indicates that providers of dementia care cannot just focus on the carer and care-recipient but need to engage with the broader care situation. Adopting a resource perspective, where potential sources of help and support can be identified, and barriers to accessing such sources considered, would be one potential approach.

Further research is needed to identify more precisely support needs in carers and how such needs might best be met, and to evaluate the effectiveness of the support offered in reducing the negative impacts of care and enhancing its positive value. Future studies should focus on specific carer groups such as spouse carers to better explore how the relationship between the PwD and the carer, the social context of the carer and care-recipient, as well as the specific disabilities brought on by dementia, affect the carer’s situation and their need for support.

## Conclusions

Spouse carers constitute over a fifth of all carers of PwD and provide more care than other carers of PwDs. Spouse carers differ from other carers in the support they provide, their caring role and the impact that providing care has on their lives. We conclude that policy-makers and practitioners must acknowledge how the experience of being a carer varies with the relationship between carer and care-recipient, so that assessments of support directed to carers are based on an understanding of the individual carer’s actual needs and preferences rather than on preconceptions of their supposed needs drawn from a generalised support model. Further research is needed to develop such support and evaluate its effectiveness.

## Supplementary Information

**Additional file 1: Apendix 1**

## Data Availability

Census data used in this study are part of Sweden’s public records managed by the official statistics authority Statistics Sweden, further information on accessing public records in Sweden can be found at https://www.scb.se/en/. The datasets used and/or analyzed during the current study are available from the corresponding author on reasonable request.

## Abbreviations

PwD: Person with dementia
NBHW: The Swedish National Board of Health and Welfare
