# Supplementary material for: A comparison of spouse and non-spouse carers of people with dementia: a descriptive analysis of Swedish national survey data
Source: BMC Geriatr. 2021 Jun 2;21:338. doi: 10.1186/s12877-021-02264-0 (PMC8170983; doi:10.1186/s12877-021-02264-0)
Supplement: Supplementary file 1 — Additional file 1: Apendix 1 [file 12877_2021_2264_MOESM1_ESM.docx]

| **Topic addressed** | **Question** | **Response Option(s)** |
| --- | --- | --- |
| Awareness of carer support legislation | |  |
|  | Before you received this questionnaire, did you know that since 2009 there has been a provision in the Social Services Act (Chapter 5, 10 §), which states that family members who care and / or support relatives shall be personally offered support from the local authority? | Yes; No |
| Care provided |  |  |
|  | Do you regularly (i.e. not occasionally or temporarily) care and/or support) someone or several people with their daily activities, personal care or in other ways due to their physical or mental illness, disability or age? | -Yes, one person  -Yes, two people  -Yes, three people  -Yes, more than three people  -No |
|  | Think about the care and support you give in total (to one or more people), how often does that occur? | -Every day  -At least every week  -At least once a month  -Less often than once a month |
|  | How many hours a week (day and night) on average do you provide care and support? | -Less than 1 hour  -1-10 hours  -11-29 hours  -30-59 hours  -60 hours or more |
| Support offered/received and /or needed | |  |
|  | Below is a list of different types of support for carers.  *Indicate for each type of support the extent to which it applies to you.*  -Information and advice  -Education  -Counselling  -Carer Support group  -Keep-fit/well activities (Nordic walking, water-gymnastics, massage or similar)  -Health check-up and health advice  -Financial benefits or financial support  -Respite (temporary formal care for the person(s) you care and support, to relieve you of your care responsibilities, whether delivered at home, via day care, or in a residential care facility  -Support via modern technology or the Internet (e.g. web information, video conferencing, Internet groups, GPS system)    -Support that facilitates work (Possibility of distance work, flexible working hours, communication with work managers via the internet or similar) | -Yes, I have been offered / received this kind of support  -No, I have not been offered / received this kind of support but would like to  -No, and I am not interested in this type of support. |
|  | Have you received any other kind of support?  -If yes, specify what: | Yes; No |
| Impact of care |  |  |
|  | Here follows a few questions about what it means for you to provide care and support. If you provide care and support to more than one person, think about all of them and all the care and support you give, regardless of whether you care and support them to different degrees.  -To provide care and support means that…  Select an answer for each statement.  ...you have trouble finding time to spend with your friends  ... you struggle to find time to exercise, for example, to take a walk or go to the gym  ... it's psychological stressful for you  ... it’s physically stressful for you  ... you experience problems in your relationship with the person(s) to whom you provide care and support  … you have financial problems  ... you experience a sense of satisfaction  ... you experience problems in your relationships with family members  …. you experience a close relationship with the person(s) to whom you provide care and support | -Always/almost always  -Often  -Sometimes  -Seldom/Never |
|  | During an average week, how much or little would you say that your sleep is disturbed due to the care and support you provide? | - My sleep is not disturbed at all  - Roughly one night per week  - Between 2 and 3 nights a week  - Between 4 and 6 nights a week  - Every night |
| Characteristics of the main care-recipient | |  |
|  | How many hours a week on average do you provide care and support to the person you care and support most? | -Less than 1 hour  -1-10 hours  -11-29 hours  -30-59 hours  -60 hours or more |
|  | Who is the person who you care and support the most? | - Husband/ wife/ partner  - Child  - Parent  - Sibling, relative  - Legal guardian, neighbour, acquaintance |
|  | In what form of housing does the person you care and support live? | - In regular accommodation (house or apartment)  - Sheltered housing  - Assisted living facility, assisted living accommodation  - Nursing home, residential care, group home |
|  | Where do you live in relation to the person you care and support? | - In the same household  - In different households but in the same building  - Within walking distance  - Not within walking distance, but less than 30 minutes one-way travel  - Between 30 minutes and one hour's one-way travel  - Between one- and three-hours one-way travel  - Between three- and five-hours one-way travel  - Over five hours one-way travel |
|  | What is the main reason why the person you care and support needs your help?  -Select only one option. | - Dementia illness, memory problems  - Neurological disability or learning difficulty (not dementia and  memory problems)  - Malignant disease, cancer or leukaemia  - Reduced physical health, impaired physical function, or physical disability  - Reduced mental health, social / personal problems, or addiction problems  - Other reason, please state: [free text] |
|  | Are there any reasons other than the one you mentioned above that this person needs your help? | - Dementia illness, memory problems  - Neurological disability or learning difficulty (not dementia and  memory problems)  - Malignant disease, cancer or leukaemia  - Reduced physical health, impaired physical function, or physical disability  - Reduced mental health, social / personal problems, or addiction problems  - Other reason, please state: |
| Main care-recipient’s need of care and support | |  |
|  | Here are some examples of activities and other things with which the person you care and support can need help.  *Select the one answer per sub-question that best describes how the person to whom you provide care and support is helped.*  -Shopping, cleaning, washing, cooking, etc.  -Practical activities such as transport, fixing things in the home and / or garden, bank and postal errands, and other services  -Physical activity, such as being encouraged to take physical exercise or being accompanied on walks  -Contacts with health and/or social care services (medical appointments, contact with home help etc.)  -Financial support / everyday expenses  -Personal care (such as hygiene, dressing and undressing, help with eating)  -Medicines and treatment (e.g., giving out medicines, wound dressing, injections)  -Supervision (looking in on, reminding, motivating, support on distance)  -Social Relationships/companionship (togetherness, stimulation, entertainment)  -Cultural activities, such as being encouraged to engage in cultural activities or being accompanied to cultural activities | - I am alone in supporting/ assisting/ providing with… [activity]…  - I support/ assist/ provide with …[activity]… with the help of other family members / friends only  - I support/ assist/ provide with …[activity]… with support from the local authority or equivalent only  - I support/ assist/ provide with …[activity]… with the help of other members of the family / friends and the local authority or equivalent  - Only other people support/ assist/ provide with …[activity]…, not myself.  - He / she does not need support/assistance regarding/ with …[activity]… |
|  | Do you think that the care and support that this person receives (i.e. from you, family / friends, local authority or other actors) is sufficient to meet his/her needs?  *Only one option can be specified. Select the best answer in your view.* | - Yes, all his/her needs for care and support are currently met  - No, and I would like to contribute more personally  - No, and I would like more help from other family members / friends  - No, and I would like more help from the local authority or equivalent  - No, and I would like more help from both family / friends and the local authority or equivalent |
| Details of participant |  |  |
|  | When were you born? | Year: |
|  | What is your current main employment?  *Only one option can be specified. Enter the option that suits you best*. | - Work as an employee [ ] % of full time  - Self-employed  - Student, intern  - On parental leave  - On leave  - Unemployment skills development programme  - Unemployed  - Retired /old age pensioner  - Sickness or activity allowance (early retirement, sickness benefit)  - Long-term sick leave (more than 3 months)  - Housewife/husband (not pensioner)  - Other, specify in the box: [free text] |
|  | How would you rate your general health condition? | - Very Good  - Pretty good  - So-so  - Pretty bad  - Very bad |
